# Teofil Simchowicz (1879–1957)

**DOI:** 10.1007/s00415-017-8460-9

**Published:** 2017-03-18

**Authors:** Andrzej Grzybowski, Aleksandra Pięta, Marta Pugaczewska

**Affiliations:** 1 Department of Ophthalmology, Poznań City Hospital, Poznan, Poland; 2 Department of Ophthalmology, University of Warmia and Mazury, Olsztyn, Poland; 3 Faculty of Medical Sciences, University of Warmia and Mazury, Olsztyn, Poland

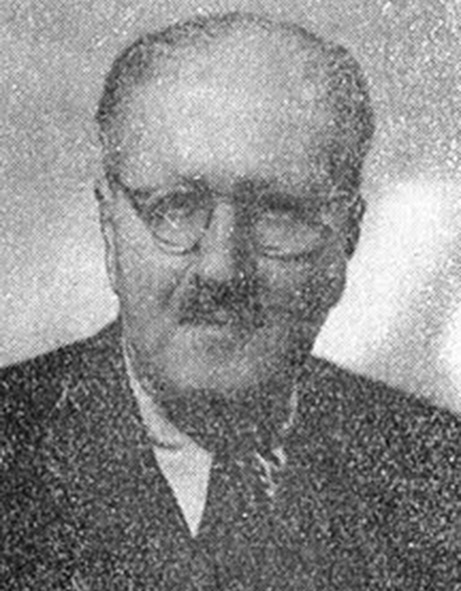



Teofil Simchowicz was a Polish-Jewish neurologist and neuropathologist interested particularly in neurodegenerative changes. He introduced to medicine the terms *senile plaques*, *granulovacuolar degeneration* and also described the nasomental reflex called *Simchowicz’s reflex*.

Teofil Simchowicz was born on June 3, 1879 in Ciechanowiec, Poland. He graduated in 1898 from VI Grammar School in Warsaw and he began his studies at the Faculty of Medicine of the Imperial University of Warsaw. He received his medical degree in 1905. In 1904 he was employed as a volunteer at the *Szpital Starozakonnych Czyste* in Warsaw, called the Jewish Hospital, where until 1911 he was an assistant at the Department of Neurology established by Edward Flatau (1868–1932)—creator of the First Polish Neurological School [1, 2].

From 1907 to 1910 Simchowicz continued his education in Germany. After six months studying in the pathological laboratory at the E. Kraepelin Department of Psychiatry in Munich he gained the ability to conduct independent research under the guidance of Alois Alzheimer (1864–1915). He described the results of the research on senile dementia in the chapter of a book by Alois Alzheimer and Franz Nissl (1860–1919) [3].

After returning to Poland, Simchowicz assumed the position of senior assistant at the Laboratory of Neurology at the Warsaw Biological Psychological Association, founded in 1911 and headed by Edward Flatau. After a few months, the laboratory was taken over by the Warsaw Scientific Society and renamed into the Department of Neurobiology, which became a part of the Marcel Nencki’s Institute of Experimental Biology established in 1912. Simchowicz investigated changes in the nervous system occurring in the course of hyperthyroidism and hypothyroidism [4].

In 1919–1921 he served in the 1st Sanitary Battalion of the Polish Army with the rank of captain doctor. After being released, he returned to the Department of Neurobiology. Due to the initiative of Edward Flatau, after his death in 1932, the Warsaw Neurobiological Institute was created. Teofil Simchowicz served as a director of the institution and the head of department of pathological anatomy up to the outbreak of the Second World War in 1939. He was co-editor and one of the editors of Polish journal on neurology *Neurologia Polska*.

He was married to Tauba Mendelsburg, their marriage was childless. In 1940 they emigrated to Palestine together. Teofil Simchowicz participated there in the life of the medical community and helped Polish refugees. In February 1944, he received a license to practice medicine in Palestine. He took part in the scientific work of Kurt Lowenstein (1883–1956) and Julius Zelermeyer (1910–2004) in the neuropsychiatric clinic Kupat Holim. He was a close friend of Leo Lipski-Lipschütz (1917–1997), Polish writer in exile.

During the last years of his life Teofil Simchowicz suffered from Parkinson disease. He died on December 31, 1957 at Hadassah Hospital in Tel Aviv [1].

Teofil Simchowicz was the author of over 20 papers published in four languages, Polish, French, German and Hebrew. In his seminal 1911 study, Simchowicz investigated 180 brain tissue samples, including patients with a clinical diagnosis of senile dementia, mentally healthy patients, patients diagnosed with atherosclerotic dementia and people affected by mental illness. The youngest subject was 63 years old, the oldest one 104 [3]. Comparatively he examined samples derived from animals, an elderly horse and two dogs (12 and 17 years old). He observed the characteristic pathological changes in people with senile dementia, which he gave the name of *senile plaques*. The plaques tend to accumulate in the cerebral cortex and form deposits encapsulated by glial cells. He concluded that in the absence of senile plaques, *dementia senilis* should be excluded, while other disorders with different clinical and anatomical picture must be considered. For the first time he described and introduced to the literature the term *granulovacuolar degeneration* specific for neurodegenerative conditions. According to the observations described by Simchowicz, these changes occur in senile dementia only in the ganglion cells of cornu Ammoni, not in the rest of the cortex. Granulovacuolar degeneration and fibrillary changes always occur simultaneously, often within the same cell.

In subsequent work he demonstrated similarities and differences between senile dementia and Alzheimer’s disease (AD) [5]. He also presented a formula called *senile index* to determine the nature of degenerative process and to evaluate its progress. He suggested that, despite the similar pathological background as in senile dementia, AD should be seen as a completely separate disease entity due to a different location of changes in the cerebral cortex, characteristic hallmarks and early onset. Location of changes explained symptoms such as receptive aphasia, apraxia and focal sings, which are not observed in dementia. In the elderly as well as in case of *dementia senilis*, plaques accumulated mainly in frontal cortex and in cornu Ammoni, rarely in parietal and temporal cortex, only a few in motor and occipital cortex. The amount of plaques coincided with expansion of cortical atrophy, therefore, Simchowicz created the *senile index*—a formula to estimate their number. According to this model the maximum quantity of plaques located in the microscope field was assessed. Fragments of cerebral cortex 20 µm thick from different parts of brain were stained using Bielschowsky or Mann–Alzheimer’s method and analyzed. In cases of AD, senile plaques occurred most frequently, according to Simchowicz, in the visual area of cortex, in contrast to the frontal predominance in the elderly and in senile dementia. For that reason the ratio of plaques seen in frontal and in occipital cortex [I.F./I.Occ.] was significant in differentiating neurodegenerative conditions such as AD and senile dementia [6].

Teofil Simchowicz’s scientific achievements include as well work on neuroendocrinology [4]. Additionally he described two reflexes: naso-ocular and nasomental [7]. The naso-ocular reflex is a physiological reflex which may disappear unilaterally in facial palsy, whereas the nasomental reflex is an intensified version of naso-ocular reflex. Simchowicz reported that nasomental reflex appears mainly in hemiparesis related to organic brain damage (unilateral) or in mental disorders and neurosis (bilateral), but it has not become a routine part of clinical examination.
